# Biochar and compost enhance soil quality and growth of roselle (*Hibiscus sabdariffa* L.) under saline conditions

**DOI:** 10.1038/s41598-021-88293-6

**Published:** 2021-04-22

**Authors:** Di Liu, Zheli Ding, Esmat F. Ali, Ahmed M. S. Kheir, Mamdouh A. Eissa, Omer H. M. Ibrahim

**Affiliations:** 1 Jiangxi Yangte River Economic Zone Research Institute, Jiujiang University, Jiujiang, China; 2 Haikou Experimental Station, Chinese Academy of Tropical Agricultural Sciences (CATAS), Haikou, China; 3 Department of Biology, College of Science, Taif University, P.O. Box 11099, Taif, 21944 Saudi Arabia; 4 Soils, Water and Environment Research Institute, Agricultural Research Center, Giza, 12112 Egypt; 5 Department of Soils and Water, Faculty of Agriculture, Assiut University, Assiut, 71526 Egypt; 6 Department of Ornamental Plants and Landscape Gardening, Faculty of Agriculture, Assuit University, Assiut, Egypt

**Keywords:** Plant stress responses, Secondary metabolism

## Abstract

Soil amendments may increase the slate tolerance of plants consequently; it may increase the opportunity of using saline water in agricultural production. In the present pot trial, the effects of biochar (BIC) and compost (COM) on roselle (*Hibiscus sabdariffa* L.) irrigated with saline water (EC = 7.50 dS m^−1^) was studied. Roselle plants were amended with biochar (BIC_1_ and BIC_2_) or compost (COM_1_ and COM_2_) at rates of 1 and 2% (w/w), as well as by a mixture of the two amendments (BIC_1_+). The experiment included a control soil without any amendments. Biochar and compost significantly enhanced the soil quality and nutrients availability under saline irrigation. Compost and biochar improved the degree of soil aggregation, total soil porosity and soil microbial biomass. BIC_1_ + COM_1_ increased the soil microbial biomass carbon and nitrogen over the individual application of each amendments and control soil. BIC_1_ + COM_1_ increased the activity of dehydrogenase and phosphatase enzymes. Growth of roselle plants including: plant height, shoot fresh and dry weight, and chlorophyll were significantly responded to the added amendments. The maximum sepal’s yield was achieved from the combined application of compost and biochar. All the investigated treatments caused remarkable increases in the total flavonol and anthocyanin. BIC_1_ + COM_1_ increased the total anthocyanin and flavonol by 29 and 17% above the control. Despite the notable improvement in soil and roselle quality as a result of the single addition of compost or biochar, there is a clear superiority due to mixing the two amendments. It can be concluded that mixing of biochar and compost is recommended for roselle plants irrigated with saline water.

## Introduction

Fresh water, of all natural resources especially in arid regions, is the major control of sustainable development. The using of salt water, with its abundance, has become an urgent matter^1^. Therefore, utilization of saline water resources to produce medicinal plants could be good strategy to address water issue^2^. In agriculture soil, salinity inhibits plant growth through osmotic effects, specific-ion toxicity and/or shortage and disorders of some nutrients^3–5^. The mechanism of combating the negative effects of salinity by adding organic matter is one of the agronomically sound practices which has been used by many researchers^6,7^. Many soil organic amendments can be used to overcome the salinity problems^8^. Organic amendments had higher CEC, water holding capacities, chelation ability, good nutrient resource, improves soil structure, aeration and its effect on soil stability^9^. The use of organic amendments to mitigate soil salinity, is cost-effective, easy-to-use techniques, an environmentally friendly method and a successful agricultural strategy^6,7^. Biochar and compost are commonly as organic amendments that can be used in this regard^10^. Organic amendments can increase the soil quality through increasing the soil nutrients availability, microbes and enzymes activity, and physiochemical properties^8,11–14^. Increasing the soil quality improves the plant growth and may increase its salt tolerance^15–17^.

Choosing the right plant in saline conditions is the most important factor in the success of the cultivation process and obtaining an economic return. Roselle (*Hibiscus sabdariffa* L.) plants are tropical wild plants and have high levels of polyphenols, anthocyanins and flavonoids which are important compounds for human health^18,19^. Roselle plants are moderately tolerance for saline and can tolerate up to 10 dS m^−1^ of water salinity^19–21^. The high levels of saline irrigation reduce the germination and vegetative growth and induce morphological, physiological and biochemical changes^20,21^.

Biochar and compost could increase soil fertility and quality and thus encourage plants to overcome the negative effects of salinity. However, little is known about the interactive effects of sole and combined application of compost and biochar on soil quality under saline conditions. Therefore, this study aims to investigate the effects of biochar and compost on soil quality and roselle growth under saline irrigation. The current study aims to investigate the following hypotheses: compared to the individual application of compost and biochar, the combined addition can assist the growth and quality of roselle through increasing the enzyme activities, soil fertility and nutrients uptake.

## Materials and methods

### Biochar and compost

Biochar was made from corn wastes by slow pyrolysis at 350 °C with a residence time of 2.5 h. Compost was made from the same corn wastes. The main characteristics of biochar were as follow: pH (11.00), EC (4.56 dS m^−1^), organic-C (520 g kg^−1^) and total N, P and K of 15, 5.40 and 30 g kg^−1^, respectively. The main characteristics of compost were as follow: pH (8.22), EC (5.25 dS m^−1^), organic-C (240 g kg^−1^) and total N, P and K of 20, 15.7 and 35 g kg^−1^, respectively.

### Pot experiment

Surface soil sample was collected from a clay loam soil and Table 1 shows some physical and chemical properties. The collected soil sample was air dried and then sieved by 2 mm sieve. Biochar and compost at rates of 1 and 2% (w/w) were mixed with the soil during the preparation of soil. The experiment included six treatments namely: control (without any amendments), biochar at two levels (BIC_1_ and BIC_2_), compost at two levels (COM_1_ and COM_2_) and BIC_1_ + COM_1_ which was a mixture of the two amendments at a rate of (1%, w/w) for each. Ten kg of the prepared soil sample were filled in black plastic pots (35 cm height and 25 cm diameter). Five seeds of roselle (*Hibiscus sabdariffa* L.cv. *Sabhia 17*) were transplanted, and after germination only two plants for each pot were left. The seed of roselle were purchased from the National Research Center, Giza, Egypt. Pots were arranged in the greenhouse in a randomized complete block design and irrigated to near filed capacity based on the weight of pots. Pots were fertilized with superphosphate (15% P_2_O_5_) at a rate of 1 g per pot which was added during the preparation of soil, as well as by 1 g N/pot from urea (46% N) three times during the experiment period. Urea and superphosphate were dissolved in water then added to the pots. During the first twenty days, the plants were irrigated with tap water, then were irrigated with underground saline water well (EC = 7.5 dS m^−1^) to the end of the experiment. At duration end, (after 150 days) plant height and the total plant fresh weight per pot were recorded. The harvested plants were washed with distilled water and oven-dried at 70 °C then the total dry matter weight per pot was estimated. Sepals were separated from the plants to record the fresh and dry weights.

**Table 1:** Some physical and chemical properties of the studied soil.

Property	Value
Sand (g kg^−1^)	250 ± 5
Silt (g kg^−1^)	390 ± 6
Clay (g kg^−1^)	360 ± 4
Texture	Clay loam
Field capacity (%w:w)	45 ± 3
Wilting point (%w:w)	20 ± 2
Bulk density (g cm^−1^)	1.40 ± 0.06
Particle density (g cm^−1^)	2.61 ± 0.08
CaCO_3_ (g kg^−1^)	15 ± 1
Organic carbon (g kg^−1^)	13 ± 1
pH (1:2)	8.15 ± 0.05
EC_e_ (dS m^−1^)	0.36 ± 0.01
CEC (cmol kg^−1^)	22 ± 2
Available—N (mg kg^−1^)	50 ± 3
Available—P (mg kg^−1^)	11 ± 0
Available—K (mg kg^−1^)	650 ± 12

Each value (± SD) is the mean of five replicates.

### Chemical analysis of biochar, compost, plant and soil

Total organic carbon (TOC) analyzer was used to measure the organic carbon content of biochar and compost. Biochar and compost samples (2.0 g) were digested with H_2_O_2_ and H_2_SO_4_^22^. The total N, P and K concentrations were measured in the digest extract. Biochar and compost pH was evaluated in a 1:5 suspension with a pH meter, and the electrical conductivity (EC) of the 1:5 extract was determined with an EC meter^23^. To measure nutrient concentrations in rosella shoots, a mixture of 7:3 ratio of sulfuric to perchloric acids was used to digest the dried ground plant material^22^. Nitrogen concentrations in the digested plant samples were measured by micro Kjeldahl’s distilling unit^22^. The method of chlorostannous and ammonium molybdate was used to measure phosphorus in the extracted plant samples which then was determined by spectrophotometer. Potassium concentrations in the plant samples extracts were measured by flame photometer^22^. Chlorophylls was determined by using SPAD 502 plus. The total anthocyanin (TAC) and flavonel (TF) was measured based on the method of Lee and Francis^23^. TAC and TF were extracted from the dried sepal’s samples by (85:15) ethanol (96%): HCl 1.5 M. The extracted solution was measured by spectrophotometer at wavelength 535 nm for TAC and 374 nm for TF.

Some physical and chemical properties of the tested soils were determined according to Burt^23^. Particle size distribution, available phosphorus, potassium and soil organic carbon (SOC) was measured as describe by^22^. Available nitrogen (NH_4_ + NO_3_) was determined using micro-kjeldahl method according to Burt^22^. Soil microbial biomass was measured by the determination of carbon and nitrogen in the soil microbial biomass (MBC and MBN) based on the method of Vance et al.^24^ and Jenkinson et al.^25^. MBC and MBN were extracted by the method of fumigation-extraction and then determined by the total organic carbon (TOC) analyzer (TOC trace, Elementar, Hanau, German). The activity of phosphatase was measured by incubation of 5 g of soil sample with 1 mL of toluene for 1 h at 37 °C as described by Guan et al.^26^. After the incubation the extract was measured by specrophtometer and expressed as g^−1^ soil h^−1^. The activity of dehydrogenase was measured by incubation of 5 g of soil sample with triphenyltetrazolium chloride for 24 h at 37 °C as described by Serra-Wittling et al.^27^. After the incubation the extract was measured by specrophtometer and expressed as g^−1^ soil h^−1^. Triphenylformazan (TPF) formed absorbance was measured by specrophtometer and expressed as mg TPF g^−1^ dry soil h^−1^. Undisturbed soil samples were collected from each pot to measure the degree of soil aggregation (DSA) and soil porosity (TSP) which are important soil quality parameters. DSA was calculated from the difference between the clay after dispersion and the clay before dispersion dived by the clay after dispersion^23^. Ring method was used to determine the soil bulk density (B_d_), while the method of density bottle was used to measure the particle density (P_d_)^23^. The following equation was used to calculate the percentage of total soil porosity (TSP):

$$ {\text{TSP}} = {1}00 \times \left( {{1} - {\text{B}}_{{\text{d}}} /{\text{P}}_{{\text{d}}} } \right), $$

where B_d_ and P_d_ is the bulk and particle density.

### Statistical analysis of data

The significance of difference between the treatments was tested by analysis of variance (one way-ANOVA). Tukey’s multiple range tests at p < 0 0.05 were performed using SPSS statistical program.

## Results

### Effects of compost and biochar on soil quality, nutrients availability and plant uptake

**Figure 1:**
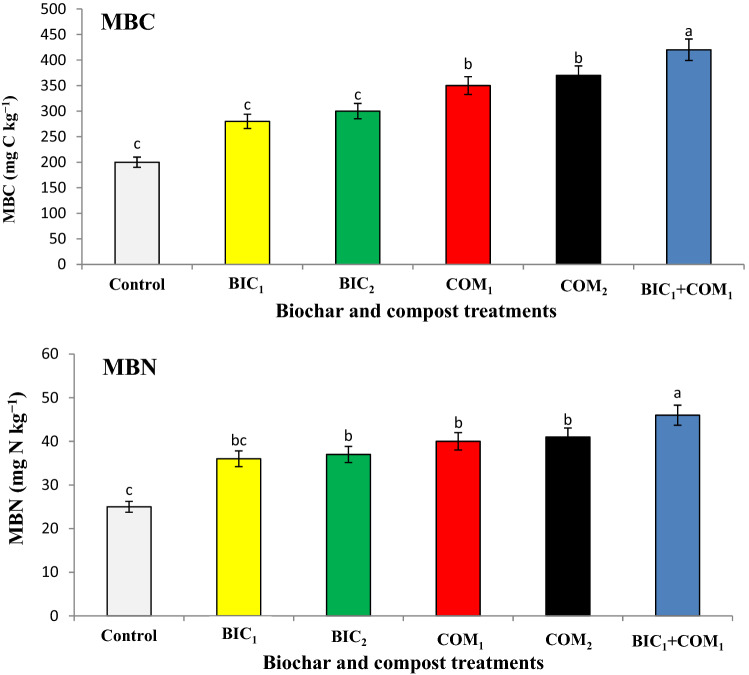
Effect of biochar and compost on soil microbial biomass carbon (MBC) and nitrogen (MBN). BIC_1_ and BIC_2_ = biochar at rates of 1 and 2% (w/w), COM_1_ and COM_2_ = compost at rates of 1 and 2% (w/w), and BIC_1_ + COM_1_ = mixture of the two amendments at 1% (w/w) of each. Means (± SD, n = 5) denoted by the same letter indicate no significant difference according to Tukey’s multiple range tests at *p* < 0.05.

**Figure 2:**
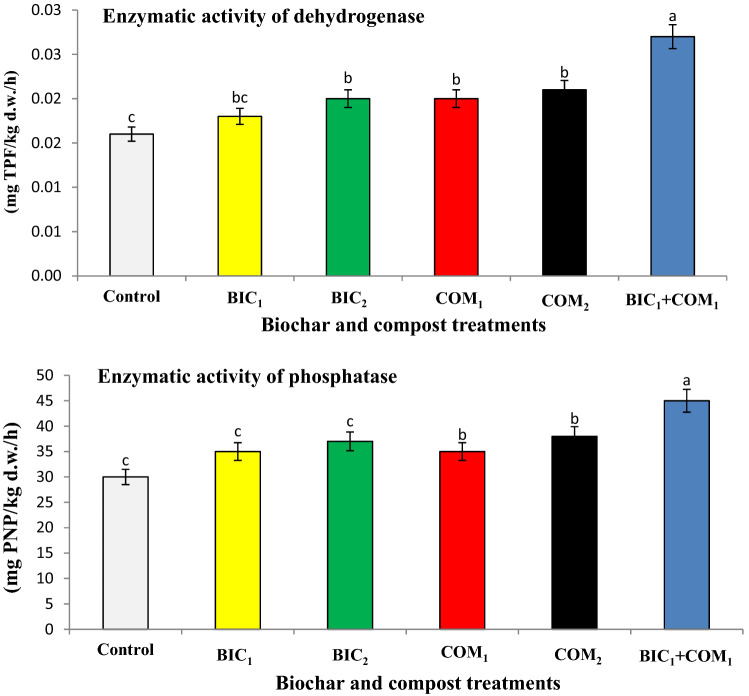
Effect of biochar and compost on enzymatic activity of dehydrogenase and phosphatase. BIC_1_ and BIC_2_ = biochar at rates of 1 and 2% (w/w), COM_1_ and COM_2_ = compost at rates of 1 and 2% (w/w), and BIC_1_ + COM_1_ = mixture of the two amendments at 1% (w/w) of each. Means (± SD, n = 5) denoted by the same letter indicate no significant difference according to Tukey’s multiple range tests at *p* < 0.05.

Addition of biochar and compost significantly affected the soil quality indicators (Table 2, Figs. 1 and 2). Biochar and compost increased the soil salinity, soil organic carbon (SOC), cation exchange capacity (CEC), total soil porosity (TSP) and degree of soil aggregate (DSA).

**Table 2:** Effect of compost and biochar on soil physiochemical properties.

	pH (1:2)	EC_e_ (dS m^-1^)	SOC (g kg^−1^)	CEC (cmol kg^−1^)	%TSP	%DSA
Control	8.10 ± 0.01a	6.0 ± 0.02b	13 ± 0 c	22 ± 1b	28 ± 2b	25 ± 2b
BIC_1_	8.25 ± 0.02a	7.4 ± 0.01a	14 ± 1b	26 ± 2a	35 ± 3a	32 ± 1a
BIC_2_	8.30 ± 0.01a	7.9 ± 0.01a	15 ± 1a	28 ± 3a	36 ± 3a	36 ± 2a
COM_1_	8.00 ± 0.00a	7.7 ± 0.01a	14 ± 1b	27 ± 2a	37 ± 3a	33 ± 3a
COM_2_	8.02 ± 0.03a	8.2 ± 0.01a	15 ± 0a	27 ± 3a	37 ± 2a	35 ± 2a
BIC_1_ + COM_1_	8.00 ± 0.01a	8.2 ± 0.01a	15 ± 1a	27 ± 2a	37 ± 2a	35 ± 3a

Means (± SD, n = 5) denoted by the same letter indicate no significant difference according to Tukey’s multiple range tests at *p* < 0.05.

*DSA* degree of soil aggregation (%), *TSP* total soil porosity (%).

The soil microbial biomass carbon (MBC) and nitrogen (MBN) affected significantly with the tested treatments (Fig. 1). The combined application of compost and biochar (BIC_1_ + COM_1_) gave the highest significant values of MBC and MBN. The maximum significant values of enzymatic activity of dehydrogenase and phosphatase were found in BIC_1_ + COM_1_, while the lowest one were found in the control soil (Fig. 2).

The findings of the present research revealed that the applying of biochar and compost showed increases in the soil available N, P and K as well as the shoot concentrations compared with the control (Table 3). Biochar and compost significantly increased N, P and K availability in the studied soil compared to the control soil. BIC_1_ + COM_1_ gave the maximum available nutrients in soil. BIC_1_ + COM_1_ increased the availability of N, P and K by 16, 38 and 15% over the control soil. Moreover, shoot concentrations of N, P and K were significantly (p < 0.05) improved by the biochar and compost additions to soil (Table 3). The combined application of compost and biochar (BIC_1_ + COM_1_) increased the concentrations of N, P and K in the shoot of roselle by 20, 31 and 25% over the control soil.

**Table 3:** Effect of biochar and compost on N, P and K availability and uptake.

Treatments	N	P	K
**Available soil nutrients (mg kg**^**−1**^**)**			
Control	70 ± 3c	16 ± 2b	650 ± 22b
BIC_1_	75 ± 4bc	17 ± 2b	750 ± 25a
BIC_2_	87 ± 3a	22 ± 2a	774 ± 27a
COM_1_	80 ± 3b	20 ± 3a	780 ± 22a
COM_2_	88 ± 4a	21 ± 3a	760 ± 21a
BIC_1_ + COM_1_	90 ± 5a	22 ± 2a	750 ± 23a
**Nutrient concentrations in plant shoots (g kg**^**−1**^**)**			
Control	25 ± 2b	4.8 ± 0.2c	15 ± 0c
BIC_1_	29 ± 2a	5.4 ± 0.2b	17 ± 1b
BIC_2_	30 ± 3a	6.5 ± 0.1a	18 ± 1a
COM_1_	28 ± 3a	6.0 ± 0.3a	16 ± 1b
COM_2_	31 ± 3a	6.2 ± 0.3a	18 ± 1a
BIC_1_ + COM_1_	30 ± 3a	6.3 ± 0.1a	18 ± 1a

Means (± SD, n = 5) denoted by the same letter indicate no significant difference according to Tukey’s multiple range tests at *p* < 0.05.

### Effects of compost and biochar on growth and yield of roselle plants

**Figure 3:**
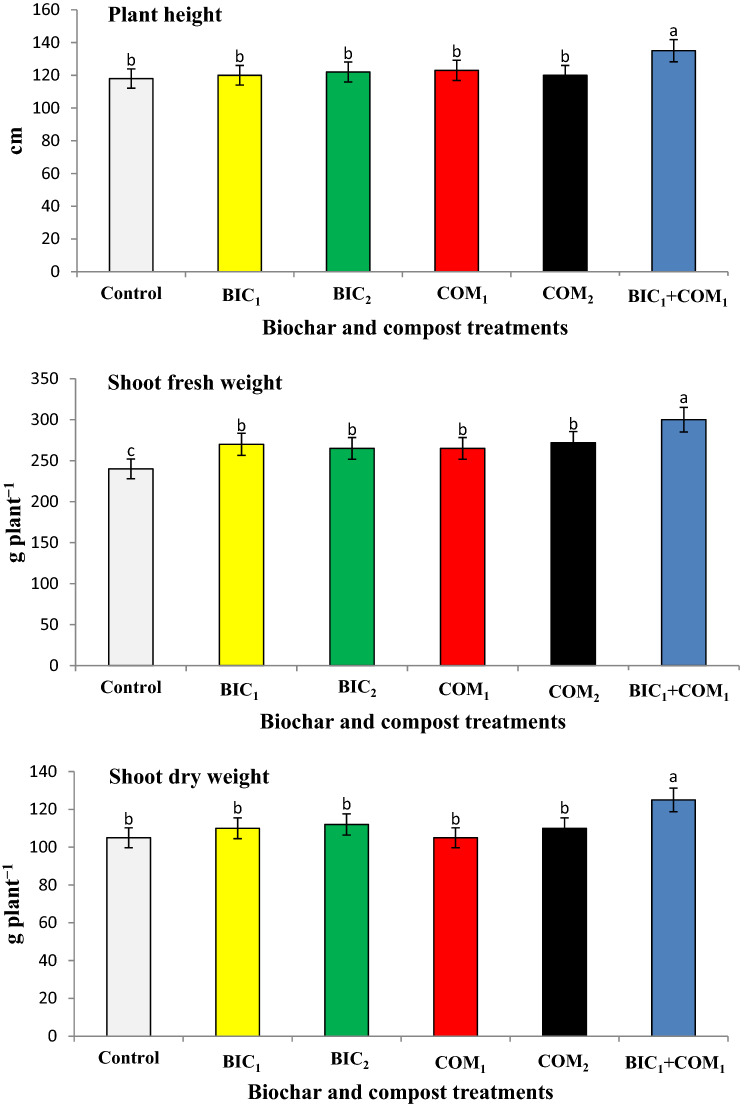
Effect of biochar and compost on some growth parameters of roselle plants. BIC_1_ and BIC_2_ = biochar at rates of 1 and 2% (w/w), COM_1_ and COM_2_ = compost at rates of 1 and 2% (w/w), and BIC_1_ + COM_1_ = mixture of the two amendments at 1% (w/w) of each. Means (± SD, n = 5) denoted by the same letter indicate no significant difference according to Tukey’s multiple range tests at *p* < 0.05.

**Figure 4:**
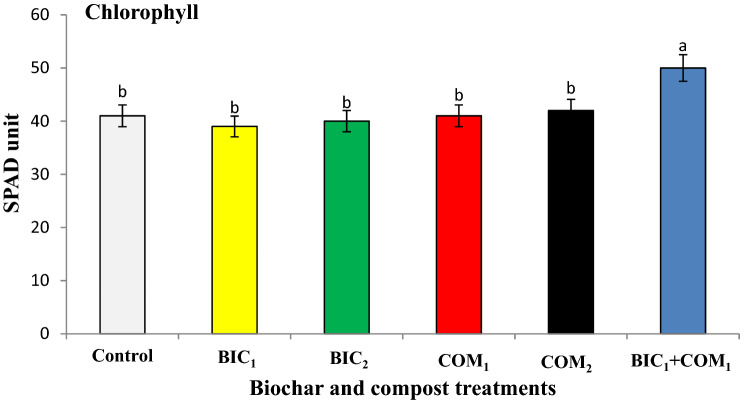
Effect of biochar and compost on chlorophyll (SPAD unit) in the leaves of roselle plants. BIC1 and BIC2 = biochar at rates of 1 and 2% (w/w), COM1 and COM2 = compost at rates of 1 and 2% (w/w), and BIC1 + COM1 = mixture of the two amendments at 1% (w/w) of each. Means (± SD, n = 5) denoted by the same letter indicate no significant difference according to Tukey’s multiple range tests at *p* < 0.05.

Response of roselle growth to the applied biochar and compost is shown in Fig. 3. The plant height, shoot fresh and dry weights significantly increased as a result of compost and biochar application. The combined application of the two amendments (BIC_1_ + COM_1_) was more effective in increasing the growth than the single application of each amendment. The combined application of biochar and compost (BIC_1_ + COM_1_) increased the plant height, dry and fresh weights by 14, 25 and 19% compared to the untreated soil. The application of BIC_1_ + COM_1_ gave the highest significant value of chlorophyll in the leaves of roselle plants (Fig. 4). BIC_1_ + COM_1_ increased the chlorophyll content by 18% compared to the control soil. Although the single addition of biochar and compost led to increases in the growth of roselle, adding the two amendments together had a superior effect in increasing growth.

**Figure 5:**
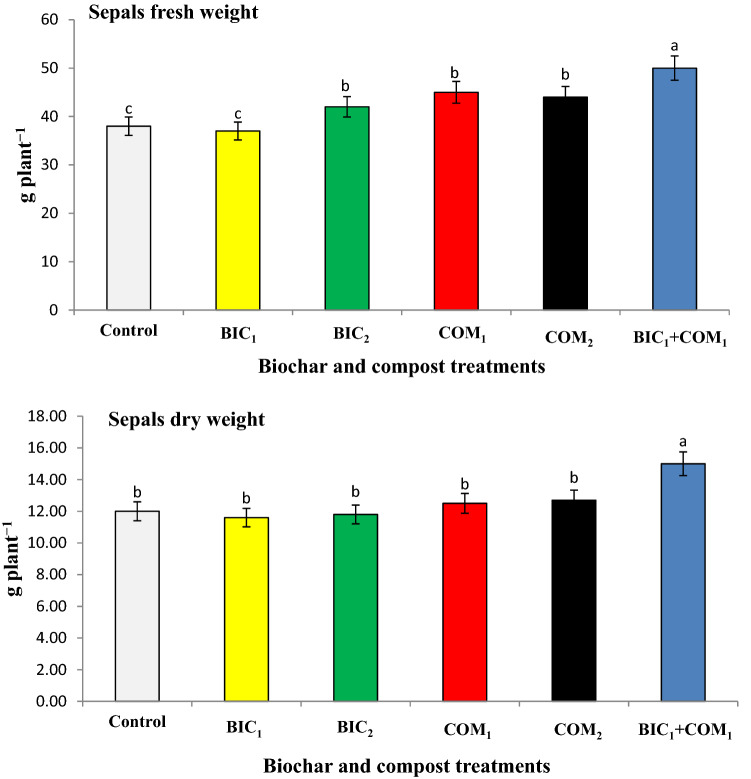
Effect of biochar and compost on the fresh (FW) and dry weight (DW) of sepal’s yield. BIC_1_ and BIC_2_ = biochar at rates of 1 and 2% (w/w), COM_1_ and COM_2_ = compost at rates of 1 and 2% (w/w), and BIC_1_ + COM_1_ = mixture of the two amendments at 1% (w/w) of each. Means (± SD, n = 5) denoted by the same letter indicate no significant difference according to Tukey’s multiple range tests at *p* < 0.05.

**Figure 6:**
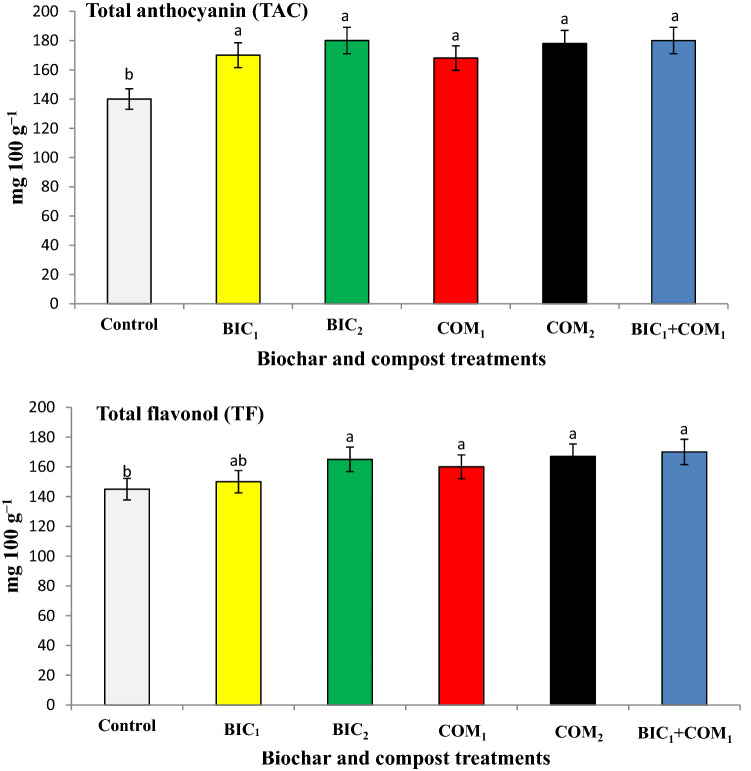
Effect compost and biochar on the total anthocyanin (TAC) and total flavonol (TF). BIC_1_ and BIC_2_ = biochar at rates of 1 and 2% (w/w), COM_1_ and COM_2_ = compost at rates of 1 and 2% (w/w), and BIC_1_ + COM_1_ = mixture of the two amendments at 1% (w/w) of each. Means (± SD, n = 5) denoted by the same letter indicate no significant difference according to Tukey’s multiple range tests at *p* < 0.05.

The fresh sepal’s yield of rosella ranged between 37 to 50 g plant ^−1^, while the dry weights of sepals ranged between 11 to 15 g plant^−1^ as shown in Fig. 5. The sepals’ fresh and dry weights were significantly increased as results of compost and biochar applications. BIC_1_ + COM_1_ increased the sepal’s fresh and dry weights by 32 and 25% above the control. The application of compost and biochar significantly increased the total flavonol (TF) and anthocyanin (TAC) above the non-amended soil (Fig. 6). BIC_1_ + COM_1_ increased the TAC and TF by 29 and 17% above the control. The combined application of both compost and biochar improved the yield and quality of roselle plants.

## Discussion

The current study clearly indicated that combined addition of biochar and compost had positive effects on soil quality and plant growth under saline irrigation conditions. The two investigated amendments enhanced the soil organic carbon, nutrients availability and improved the soil aggregation and porosity. Increasing the soil organic carbon through the application of biochar and compost caused an increasing the activity of soil microbes that increased the nutrients release and enhanced soil physiochemical characteristics e. g., the water holding, CEC and soil structure^28–30^. The combined application of both compost and biochar increased the soil microbial biomass carbon and nitrogen over individual treatments. Biochar and compost have great effects on soil biological and physiochemical characteristics^12,31,32^. Mahmoud et al.^14^ studied the effect of compost and biochar on the quality of metal polluted soil and they found that the soil microbial biomass carbon increased as results of compost and biochar together than the single application of each amendment. The combined addition of compost and biochar is more effective in increasing soil productivity and soil quality^14^. The soil that was amended with compost and biochar exhibited high CEC and SOC than the control soil. Improvement of soil CEC and SOC may be due to the functional groups (e.g. hydroxyl and carboxylate) in compost and biochar^14,33,34^ or due to the release of low-molecular weights of organic substances as results of mineralization of the added organic amendments^14,35^.

The application of biochar and compost caused remarkable increases in the yield and quality of several field and vegetable crops through increasing the soil organic matter, nutrients availability and plant uptake^30,32,36,37^. The results of the current study revealed that there were increases in the soil microbial biomass carbon and nitrogen associated with the application of biochar and compost. Biochar and compost are rich with organic matter which will encourage the growth of many benefit microorganisms and these organisms have a good ability to produce various organic acids compounds that helping in nutrients availability or promoting plant growth^38–41^. The activity of soil microbes can be evaluated through the measuring of soil microbial biomass carbon and nitrogen. The activity of soil microbes is an indicator for the decomposition of any added organic residues to soil and the rates of nutrients release^14,42^. The mixture of compost and biochar gave the highest value of MBC and MBN in this study as well as the activity of dehydrogenase and phosphatase enzymes. Biochar and compost mixture enhanced the soil nutrient availability and increased the population of soil microbes^13,43^. Increasing the microbial activity enhanced the enzyme activity, which increase the plant nutrients uptake and growth^15–17^. Remarkable increase in the rosella growth was observed with the additions of biochar and compost. This result could be referring to the improvement in physical and chemical properties^44^. Addition of biochar and compost to saline irrigated plants lessened the negative impacts of salts and enhanced the growth of plants by increasing the essential nutrients release from the added organic material which may help to balance the negative adverse of salinity^8,45–47^.

Biochar is characterized by its higher content of more stable organic carbon compounds compared to compost, and thus it slowly decomposes in the soil^34,36^, thus, it becomes more effective in improving the soil physiochemical properties^36^. The decomposing organic materials in compost encourage the growth of soil microorganisms and increase the activity of soil enzymes^43,48^. The integrated effect of compost and biochar improves the physicochemical properties of soil and increases the activity of soil enzymes and microorganisms better than the single additives^33,34^.

## Conclusions

The response of saline irrigated roselle to the individual and combined application of compost and biochar was investigated in a pot experiment. The individual addition of compost or biochar increased the soil quality indicators but the combined application of the two amendments exhibited a superiority in that respect. Increasing of soil quality caused remarkable increases in the growth and yield of roselle plants under saline conditions. The activities of soil microbes and enzymes were improved due to the application of the two amendments together. The findings of this research showed that the application of compost and biochar increased the ability of roselle plants to tolerate saline irrigation. According to this study, marginal water can be used to irrigate roselle plants with applying of both compost and biochar.
